# Regulation of K^+^ Nutrition in Plants

**DOI:** 10.3389/fpls.2019.00281

**Published:** 2019-03-20

**Authors:** Paula Ragel, Natalia Raddatz, Eduardo O. Leidi, Francisco J. Quintero, José M. Pardo

**Affiliations:** ^1^ Instituto de Bioquímica Vegetal y Fotosíntesis, Consejo Superior de Investigaciones Científicas y Universidad de Sevilla, Seville, Spain; ^2^ Centre for Organismal Studies, Universität Heidelberg, Heidelberg, Germany; ^3^ Instituto de Recursos Naturales y Agrobiologia de Sevilla, Consejo Superior de Investigaciones Cientificas, Seville, Spain

**Keywords:** plant nutrition, potassium, nitrate, regulation, long-distance transport

## Abstract

Modern agriculture relies on mineral fertilization. Unlike other major macronutrients, potassium (K^+^) is not incorporated into organic matter but remains as soluble ion in the cell sap contributing up to 10% of the dry organic matter. Consequently, K^+^ constitutes a chief osmoticum to drive cellular expansion and organ movements, such as stomata aperture. Moreover, K^+^ transport is critical for the control of cytoplasmic and luminal pH in endosomes, regulation of membrane potential, and enzyme activity. Not surprisingly, plants have evolved a large ensemble of K^+^ transporters with defined functions in nutrient uptake by roots, storage in vacuoles, and ion translocation between tissues and organs. This review describes critical transport proteins governing K^+^ nutrition, their regulation, and coordinated activity, and summarizes our current understanding of signaling pathways activated by K^+^ starvation.

## Introduction

Potassium (K^+^) is of paramount importance in plant cell physiology. K^+^ is an essential macronutrient that fulfills critical functions related to enzyme activation, osmotic adjustment, turgor generation, cell expansion, regulation of membrane electric potential, and pH homeostasis (Hawkesford et al., 2012). While the K^+^ concentration in the soil solution may vary widely from 0.01 to 20 mM, plant cells maintain a relatively constant concentration of 80–100 mM in the cytoplasm (Rodriguez-Navarro, 2000). Moreover, plants accumulate large amounts of K^+^ in their vacuoles, surpassing purely nutritional requirements. Hence, K^+^ is the most abundant cation in plant cells, comprising up to 10% of plant dry weight and often exceeding the ca. 2% that supports near-maximal growth rates (White and Karley, 2010). There is a steep curvilinear relationship between the tissue concentration of K^+^ and plant growth, from which a critical concentration of K^+^ supporting 90% of maximum yield can be determined. Above this concentration, growth has no correlation with the increased K^+^ content, but at lower K^+^ concentrations, growth declines rapidly. Consequently, K^+^ fertilization is common practice in modern agriculture and about 40–60% of crop yields are attributable to commercial fertilizer use (Stewart et al., 2005). However, agricultural fertilization is far from being fine-tuned with nutritional requirements.

**Figure 1:**
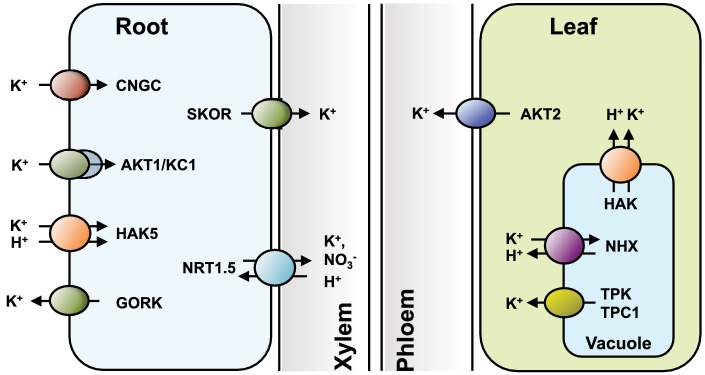
Transporters involved in K^+^ uptake by roots and inter-organ partition. HAK5, AKT1, and non-selective cyclic nucleotide-gated cation channels (CNGC) all contribute to K^+^ nutrition, albeit at different ranges of substrate concentrations, from low- to high-availability, respectively. K^+^ efflux through the outward-rectifying GORK channel facilitates the fine-tuning of plasma membrane electrical potential, and allows repolarization under circumstances that promote depolarization, such as salinity stress. In the root stele, the outward-rectifying SKOR channel releases K^+^ into the xylem vessels for nutrient delivery to the shoots. The nitrate transporter NRT1.5 facilitates K^+^ uploading into the xylem either by electrical coupling with other K^+^-selective transporters or directly acting as K^+^/H^+^ antiporter. In aerial tissues, an array of K^+^-influx channels and KT/HAK/KUP carriers allow the uptake of the incoming K^+^ into green cells. K^+^ is stored inside vacuoles by NHX exchangers and released back to the cytosol by TPK and TPC1 channels, and possibly also by KT/HAK/KUP carriers at the tonoplast (the vacuole in root cells is omitted for simplicity). The plasma membrane outward K^+^ channel AKT2 releases K^+^ into the phloem for returning K^+^ to the root and to facilitate the uploading of photosynthates into the phloem sap.

K^+^ is taken up from the soil solution by root epidermal and cortical cells. Once K^+^ is inside the root symplast, it may be stored in vacuoles, where it fulfills osmotic functions, or is transported to the shoot *via* xylem (Pardo and Rubio, 2011). In turn, shoot cells may also supply stored K^+^ for redistribution *via* phloem. In this transit from the soil to the different plant organs, K^+^ crosses various cell membranes through K^+^-specific transport systems (Figure 1). Coordinated operation of the different transport systems within the plant to secure K^+^ uptake from the soil and delivery to the different plant organs requires complex K^+^ sensing and signaling mechanisms. Because of the extraordinary diversity of K^+^ transporters in plant cells and the physiological and developmental processes in which they are involved, this review is focused on the molecular mechanisms mediating K^+^ uptake and release at the plasma membrane level, with an emphasis on K^+^ absorption from the soil and distribution throughout the plant due to the relevance of these processes in plant nutrition. Storage of K^+^ into vacuoles is treated only briefly and readers are referred to other comprehensive reviews describing transport systems operating at the tonoplast (Martinoia et al., 2012; Ahmad and Maathuis, 2014; Eisenach and De Angeli, 2017; Martinoia, 2018). Last, because of the extensive interactions of nitrogen and K^+^ in plant mineral nutrition, we summarize the coordinated regulation of NO_3_^−^ and K^+^ uptake and long-distance transport in *Arabidopsis*.

Uptake and distribution of K^+^ in plant cells is carried out by a variety of transporter proteins categorized into several families with varied structures and transport mechanisms that comprise the channel families *Shaker*-like voltage-dependent, the tandem-pore (TPK), and the two-pore channels (TPC) (Hedrich, 2012), the carrier-like families KT/HAK/KUP (Nieves-Cordones et al., 2014a; Li et al., 2018), HKT uniporters and symporters (Hamamoto et al., 2015), and cation-proton antiporters (CPA). The CPA family is the largest one and includes the NHX, CHX, and KEA antiporters (Sze and Chanroj, 2018). In this review, we describe the structure and diversity of the main K^+^ transporter families whose members contribute substantially to K^+^ nutrition. Other proteins with uncertain roles or descriptions of transport activities without candidate proteins have been omitted.

## Transport Protein Families Involved in K^+^ Nutrition

### K^+^-Selective Channels

**Figure 2:**
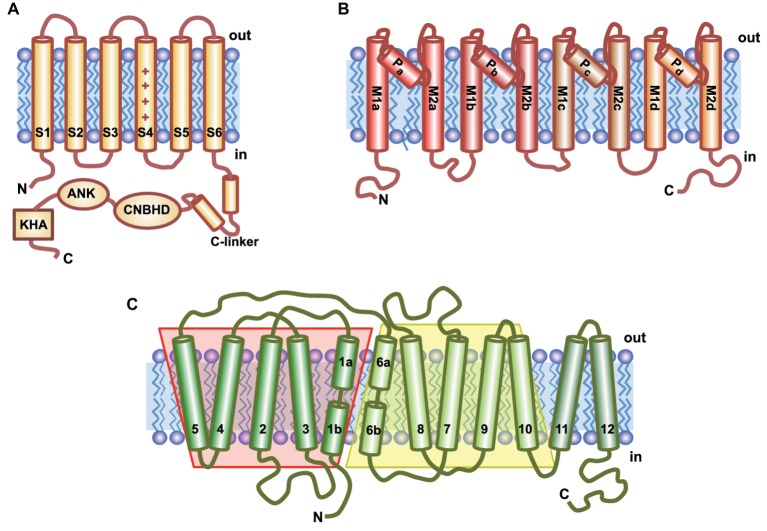
Topological models of the main ion transporters involved in K^+^ nutrition. **(A)** Voltage-gated K^+^ channels contain six transmembrane domains (S1–S6); S4 is the voltage-sensor characterized by the array of positively charged amino acids (+). The long C-terminal tail contains several conserved domains: C-linker, a cyclic nucleotide binding homologous domain (CNBHD), an ankyrin domain (ANK), and a final region rich in hydrophobic and acidic residues (KHA). **(B)** HKT transporters have a channel-like structure that contains four identical subunits (a–d), each comprising two transmembrane helices (M1 and M2) connected by the P-loop involved in ion selectivity. **(C)** KT/HAK/KUP transporters have 12 putative transmembrane domains (TMs). TM1-5 and TM6-10 are predicted to fold in the same conformation but showing inverse symmetry.

The first K^+^ transporter with a role in nutrient uptake was the *Shaker*-like, voltage-gated, and K^+^-selective channel AKT1 (Hirsch et al., 1998). Although voltage-gated (VG) channels of plants are phylogenetically related to animal *Shaker* channels, they are distinct and include additional functional domains (Jegla et al., 2018). The basic architecture of VG channels consists of four α-subunits surrounding a central aqueous pore for K^+^ permeation. Each subunit contains six transmembrane segments, named S1–S6, which can be divided into two different modules: the first four α-helices form a voltage-sensor domain that contains multiple positively charged residues that moves within the membrane in response to voltage. This movement is directly coupled to the opening or closing of the channel. The segments S5, S6, and the pore loop, form the pore domain, named P, where each of the four subunits contributes equally to the permeation pathway. Moreover, plant α-subunits have a long C-terminal region constituting more than half of the protein (Figure 2). This cytosolic tail includes several functional domains: (1) a linker region (C-linker) proximal to the pore that transduces conformational changes that gate the channel and that may also determine the target membrane (Nieves-Cordones et al., 2014b; Jegla et al., 2018); (2) a conserved and essential cyclic nucleotide-binding homology domain (CNBHD) whose function is not the binding of cNMP but to mediate the interactions between subunits within the channel tetramer; (3) an ankyrin domain (found in only six out of the nine *Arabidopsis* VG channels), which may mediate the binding of interacting proteins (Michaely and Bennett, 1992); and (4) a distal KT/KHA domain rich in hydrophobic and acidic residues, that is unique to plant K^+^ channels, and is involved in channel tetramerization and clustering at the membrane (Daram et al., 1997; Ehrhardt et al., 1997; Zimmermann et al., 2001; Dreyer et al., 2004).

Plant voltage-gated K^+^ channels are divided into three subfamilies regarding their response to the membrane potential (Dreyer and Uozumi, 2011): (1) Inward-rectifying (Kin) channels that in *Arabidopsis* include AKT1, AKT6, KAT1, and KAT2; they open at hyperpolarized membrane potentials allowing the uptake of K^+^. (2) Outward-rectifying (Kout) channels that mediate K^+^ release because they open at depolarized membrane potentials; this group is composed of SKOR and GORK channels. (3) Weakly rectifying (Kweak) channels that can mediate both K^+^ uptake and release, and whose *Arabidopsis* representative is AKT2. In addition, the *Arabidopsis* KC1 (KAT3) is an electrically silent *Shaker*-like protein that interacts with and regulates functionality of the Kin channels AKT1, KAT1, KAT2, and AKT2, but not the Kout channels (Jeanguenin et al., 2011). This interaction negatively shifts the activation threshold of Kin channels and decreases the macroscopic inward conductance compared to that of homomeric channels (Dreyer et al., 1997; Duby et al., 2008; Geiger et al., 2009). Heteromerization of different subunits of Kin channels is of great importance to increase the functional diversity and regulation of different cell types (Dreyer et al., 1997; Véry and Sentenac, 2003; Xicluna et al., 2007; Jeanguenin et al., 2008; Lebaudy et al., 2008; Lebaudy et al., 2010). Although this behavior has also been suggested for outward-rectifier (Kout) channels, heteromerization has been reported only among subunits of Kin or Kout, preventing formation of heteromeric structures between the two subunit types (Dreyer et al., 2004).

### K^+^-Uptake Carriers

Proteins of the KT/HAK/KUP family are present in plants, fungi, bacteria, and even viruses (Greiner et al., 2011; Santa-Maria et al., 2018), and they are often associated with K^+^ transport across membranes and K^+^ supply. In bacterial genomes, K^+^ carriers of this family are encoded by single-copy genes named *kup*. In *Escherichia coli*, kup is a constitutive low-affinity uptake system that operates as K^+^-H^+^ symporter (Zakharyan and Trchounian, 2001). In fungi, the homologous proteins are encoded by *HAK1*-like genes present as one- or two-copy in most species. In contrast to bacterial *kup*, fungal *HAK* genes are strongly induced by K^+^ starvation and the encoded proteins mediate high-affinity K^+^ transport (Benito et al., 2011). In plants, these transporters are known as KT, HAK, or KUP (KT/HAK/KUP family) and they are represented by multiple genes in their genomes. Members of this family have been widely associated with high-affinity K^+^ uptake from the soil, while others may function in both low-affinity and/or high-affinity transport (Luan et al., 2009; Very et al., 2014) and other roles related, for example, to K^+^ translocation, control of water movement at the plant level, salt tolerance, osmotic/drought responses, transport of other alkali cations, and developmental processes in plants, such as root hair growth and auxin distribution (Li et al., 2018; Santa-Maria et al., 2018). These diverse functions of KT/HAK/KUP transporters may all result from their critical roles in cellular K^+^ homeostasis. *KT/HAK/KUP* genes are not present in animal cells, what could indicate that they are crucial for K^+^ transport in organisms facing external solutions with fluctuating and very low K^+^ concentrations, often in the μM range (Ashley et al., 2006). Based on the present knowledge, *KT/HAK/KUP* genes are present in all plant genomes, which contrasts with that in the other kingdoms, where they are only present in certain species (Grabov, 2007; Greiner et al., 2011). This difference may reflect the importance of these transporters for the plant’s way of life.

KT/HAK/KUP transporters of land plants are classified according to their sequence homology into six clusters or clades (I–VI), with clade VI including only members of bryophytes (Santa-Maria et al., 2018). Phylogenetic analysis shows that all KT/HAK/KUPs from algae diverge from land plant clades, suggesting that the diversification into these groups took place after the colonization of land by green organisms (Santa-Maria et al., 2018). The KT/HAK/KUP group from angiosperms displays a high and rather variable number of members in the different plant species genomes that have been sequenced so far. For instance, there are 13 genes in *Arabidopsis*, 16 in peach, 17 in grapevine, 20 in *Medicago*, 21 in *Cassava*, 27 in rice, maize, and *Brachypodium,* and 57 in *Panicum virgatum* (Song et al., 2015; Nieves-Cordones et al., 2016a,c; Ou et al., 2018). Members of KT/HAK/KUP family in angiosperm are classified among clades I–V (Nieves-Cordones et al., 2016c).

The KT/HAK/KUP transporters involved in K^+^ uptake from the soil are clustered into a distinct subgroup of clade I, termed Ia (Nieves-Cordones et al., 2016c) and that we call herein HAK1-like transporters by analogy with the fungal counterparts. This subgroup includes barley HvHAK1 (Santa-María et al., 1997; Gierth and Mäser, 2007), *Arabidopsis* AtHAK5 (Rubio et al., 2000; Gierth et al., 2005), rice OsHAK1 and OsHAK5 (Bañuelos et al., 2002), pepper CaHAK1 (Martinez-Cordero et al., 2004), tomato LeHAK5 (Nieves-Cordones et al., 2007), and *Thellungiella* ThHAK5 (Alemán et al., 2009b). High-affinity K^+^ transport has been demonstrated for all the HAK1-like transporters in heterologous expression systems (Nieves-Cordones et al., 2014a). On the other hand, AtKUP7, belonging to clade V, could be involved in K^+^ uptake from low to moderate external K^+^ concentrations (Han et al., 2016), and thus the participation in K^+^ uptake from soil of proteins from different clades should not be discarded. Interestingly, other members of clade I could be related with K^+^ uptake by cells from specialized tissues. DmHAK5 from Venus flytraps is implicated in the uptake of K^+^ released from the digested prey in the bi-lobed capture organ (Scherzer et al., 2015), whereas the quinoa CqHAK5-like drives K^+^ influx into cells of the leaf salt bladders to contribute to the osmotic balance of the cytosol against the osmotic pressure of the salt-containing vacuoles (Bohm et al., 2018).

KT/HAK/KUP transporters are phylogenetically related to the superfamily of acid-polyamine-organocation (APC) transporters that comprises secondary active transport proteins responsible for uniport, symport, and antiport of a wide range of substrates (Vastermark et al., 2014). Taking as template crystal structures of prokaryotic APC transporters, computational 3D modeling of AtKUP7 (Ahn et al., 2004; Al-Younis et al., 2015; Santa-Maria et al., 2018), AtKUP4/TRH1 (Daras et al., 2015), OsHAK1 (Rai et al., 2017), AtKUP1/TRH1 (Santa-Maria et al., 2018), AtHAK5 (Santa-Maria et al., 2018), and HvHAK1 (Santa-Maria et al., 2018) has been reported. The structural models (Figure 2) show the presence of common attributes among all of them: (1) a hydrophobic core containing 10–14 transmembrane (TM) segments; and (2) three cytosolic domains—the N- and C-termini and a region containing approximately 70 residues situated between second and third TMs (loop II–III). Although, the structure of the pore region has not been described yet, several works have analyzed the effect of mutations on the function of these transporters (Santa-Maria et al., 2018). So far, results indicated that several parts of the protein may contribute to setting the Vmax of the transporter and that the region including from N-terminus to loop II–III may contribute in determining its Km. Furthermore, sequence alignments show that, although there is not extensive sequence conservation, 40 amino acid residues are conserved in exactly the same position in all the eukaryotic HAK transporters and in the Kup bacterial transporters (Rodriguez-Navarro, 2000). Six of these conserved residues are included in a highly conserved motif in the first transmembrane domain whose consensus sequence is **G**VVY**GD**LGTS**PLY** (the amino acids conserved in all proteins are in bold) (Rodriguez-Navarro, 2000). A helical-wheel representation of this transmembrane fragment locates three glycine residues on the same side of the helix, which in the case of a tetrameric structure may operate as a substrate selectivity filter analogous to the GXGYGD motif highly conserved in K^+^ channels. Regarding this, it has been suggested that AtKUP4/TRH1 may form homodimers (Daras et al., 2015), likely involving the interaction between C-terminus domains and less likely between loops II–III.

Contrary to VG channels that are all targeted to the plasma membrane, KT/HAK/KUP transporters have been reported in different subcellular compartments (Table 1). The majority of the characterized transporters of the KT/HAK/KUP family are located in the plasma membrane, although not all of them are involved in K^+^ nutrition. For instance, AtKUP4/TRH1 seems to participate in auxin transport related with root gravitropism and root hair development (Rigas et al., 2013), whereas AtKUP6 acts in lateral root initiation and development in the auxin and ABA crosstalk signaling pathways (Osakabe et al., 2013).

**Table 1:** Sub-cellular location of selected *Arabidopsis*, barley, rice, and *Physcomitrella patens* KT/HAK/KUP transporters.

Transporter/location	Plasma membrane	Tonoplast	ER-like membranes	Thylakoid membranes	References
AtHAK5	√				Qi et al., 2008
AtKUP4/TRH1	√	√	√		Rigas et al., 2013
AtKUP5		√			Jaquinod et al., 2007; Whiteman et al., 2008
AtKUP6	√				Osakabe et al., 2013
AtKUP7	√	√			Han et al., 2016
AtKUP8		√			Jaquinod et al., 2007; Whiteman et al., 2008
AtKUP12		√		√	Kleffmann et al., 2004; Jaquinod et al., 2007; Whiteman et al., 2008
HvHAK1	√				Senn et al., 2001
OsHAK1	√				Chen et al., 2015
OsHAK5	√				Yang et al., 2014
OsHAK10		√			Bañuelos et al., 2002
OsHAK21	√				Shen et al., 2015
PpHAK2			√		Haro et al., 2013
PpHAK3			√		Haro et al., 2013

### HKT Proteins

The ample repertoire of transporters encoded in the genome of plants includes proteins that are collectively known as High affinity K^+^ Transporters (HKTs; Figure 2) despite the fact that these proteins facilitate Na^+^-selective uniport or Na^+^-K^+^ symport with a channel-like activity (Benito et al., 2014). Phylogenetic and functional analyses distinguished two HKT subfamilies (Platten et al., 2006). Members of subfamily I (HKT1) are ubiquitous in plants, Na^+^-selective, and mostly involved in Na^+^ recirculation through vascular tissues, as best exemplified by AtHKT1;1 (Sunarpi et al., 2005). Members of subfamily II (HKT2) have been found only in monocotyledonous species. Although they are all K^+^-permeable, mechanistically HKT2s can operate as either Na^+^-K^+^ symporters or K^+^-selective uniporters [reviewed by Benito et al. (2014)]. HKT2-like proteins of cereals have been involved in K^+^ nutrition.

## K^+^ Uptake by Roots

The uptake of K^+^ by roots (often measured with rubidium as tracer) exhibits a biphasic kinetics in response to increasing external concentrations corresponding to high- and low-affinity transport systems, which work at low (<1 mM) and high (>1 mM) external K^+^ concentrations respectively (Epstein et al., 1963; Gierth and Mäser, 2007). At high concentration in the soil solution, K^+^ crosses the membrane mostly through channels. The channels simply give a path for the ions allowing them to move down the electrochemical gradient. At low K^+^ concentration, active transporter systems are needed in order to pull K^+^ inside the cell against its electrochemical gradient. However, studies in several plant species have shown that channels may be involved in K^+^ uptake in the high-affinity range of K^+^ concentrations (Rubio et al., 2010) as long as the membrane is sufficiently hyperpolarized, i.e. highly electronegative inside (Hirsch et al., 1998; Spalding et al., 1999; Gierth and Mäser, 2007; Rubio et al., 2010).

The sensitivity to NH_4_^+^ is an important characteristic of high-affinity K^+^ uptake mediated by carriers that has been used as a tool for the identification of additional high-affinity transport systems (Santa-María et al., 1997; Nieves-Cordones et al., 2007). NH_4_^+^-sensitive and -insensitive components of high-affinity K^+^ uptake have been identified in *Arabidopsis* (Spalding et al., 1999), barley (Santa-Maria et al., 2000), pepper (Martinez-Cordero et al., 2005), and rice (Bañuelos et al., 2002; Chen et al., 2015). Results indicate that the NH_4_^+^-sensitive component of K^+^ uptake is likely mediated by KT/HAK/KUP transporters (HAK1-like transporters), whereas inward-rectifier K^+^ channels (AKT1-like channels) constitute the NH_4_^+^-insensitive pathway (Santa-Maria et al., 2000; Nieves-Cordones et al., 2014a). Together, AKT1-like channels and HAK1-like transporters are now thought to constitute the main systems for K^+^ uptake in plants under low-K^+^ concentrations (Table 2). However, the NH_4_^+^-sensitive and -insensitive pathways appear to contribute differently to high-affinity K^+^ uptake depending on the plant species and the ionic external concentration of transported substrates, mainly K^+^, NH_4_^+^, and Na^+^ (Aleman et al., 2011; Nieves-Cordones et al., 2016c).

**Table 2:** Comparison of AKT1 channels and HAK1/HAK5 transporters from *Arabidopsis* and rice working at different ranges of external K^+^ concentrations. The uptake systems working in addition to AKT1 and HAK1/HAK5 likely include CHX exchangers (Zhao et al., 2008) and cyclic nucleotide-gated channels (CNGC) that may contribute to K^+^ absorption when the external K^+^ concentration is sufficiently high (Caballero et al., 2012).

External [K^+^]	Arabidopsis	Rice
<10 μM	AtHAK5	OsHAK1
10–100 μM	AtHAK5 AtKUP7 AtAKT1	OsHAK1 OsHAK5 OsAKT1
100–200 μM	AtHAK5 AtAKT1	OsHAK1 OsHAK5 OsAKT1
200 μM–1 mM	AtAKT1	OsHAK1 OsHAK5 OsAKT1
1–10 mM	AtAKT1 Other systems (CHX, CNGC)	OsAKT1 Unknown systems
>10 mM	Other systems (CHX, CNGC)	Unknown systems

Among the inward-rectifying K^+^ channels of *Arabidopsis*, only AKT1 and AtKC1 are abundantly expressed in root tissues (Reintanz et al., 2002). AtKC1 expressed alone remains in the endoplasmic reticulum, but it can be recruited to the plasma membrane to regulate AKT1 activity (Duby et al., 2008; Geiger et al., 2009; Honsbein et al., 2009; Wang et al., 2010). In addition, AKT1 is positively regulated by the protein kinase complex comprising the kinase CIPK23 and one of the two alternative calcium-dependent regulatory subunits CBL1 and CBL9 (Li et al., 2006; Xu et al., 2006). AKT1 possesses an intrinsic K^+^ sensor reducing channel conductance at submillimolar external K^+^ concentrations. Despite this K^+^ sensor, upon activation by the CIPK/CBL complex at low external K^+^, the homomeric AKT1 channels open at voltages positive of *E*_K_, a condition potentially resulting in cellular K^+^ leakage (Geiger et al., 2009). Incorporation of the *At*KC1 subunit into the channel complex, however, shifts the voltage dependence of AKT1 toward more negative potentials (ca. −70 mV) to prevent K^+^ loss (Geiger et al., 2009; Wang et al., 2010; Wang et al., 2016). In other words, AKT1/KC1 heteromerization renders the channel more efficient at blocking K^+^ permeation in the outward direction. The physical interaction of the CIPK23/CBL1 complex is specific for AKT1 channels and does not involve the AtKC1 subunit. The gain-of-function mutation *AtKC1-D* (G322D substitution in transmembrane S6) was recovered in the *cipk23* mutant background. *AtKC1-D* enhanced the inhibition of AKT1 channel activity and restricted K^+^ leakage through AKT1 under low-K^+^ conditions, thereby increasing the tolerance to nutrient stress (Wang et al., 2016). Although the double mutant *akt1 KC1-D* was sensitive to low-K^+^, indicating that KC1-D action is through AKT1, an additional indirect effect of mutation KC1-D through HAK5 cannot be ruled out. By inhibiting AKT1 and shifting its voltage dependence toward a more negative direction, the plasma membrane could become hyperpolarized in the *KC1-D* mutant, thereby enhancing the expression and activity of HAK5 and improving net K^+^ uptake.

Several mechanisms for AKT1 deactivation have been proposed. The PP2C-type protein phosphatase AIP1 interacts with and inactivates the AKT1 channel, counteracting the activation by CIPK23 in oocytes (Lee et al., 2007). In principle, these findings are evidence of a phosphorylation/dephosphorylation switch that regulates AKT1 channel activity, but it should be noted that no phosphorylation of AKT1 by CIPK23 and dephosphorylation by AIP1 has been demonstrated conclusively (Hashimoto et al., 2012). Instead, four components, CIPKs, CBLs, PP2Cs, and AKT1, appear to interact mutually and form a molecular complex whose specific composition could ultimately regulate channel activity (Lan et al., 2011). In this model, PP2C phosphatases interact with the kinase domain of CIPKs to counteract kinase-mediated activation of AKT1. Upon calcium signaling, CBLs interact with PPC2C to inhibit their phosphatase activity while simultaneously activating the partnering CIPKs. On the other hand, CBL10, a regulatory subunit of CIPK24/SOS2 but not of CIPK23, also interacts directly with AKT1 and negatively modulates AKT1 activity by competing with CIPK23 to bind AKT1 (Ren et al., 2013). Since CBL10 function is related to salinity stress rather than to mineral nutrition (Kim et al., 2007; Quan et al., 2007; Lin et al., 2009), this cross-regulation may constitute a mechanism to prevent salinity-induced K^+^ loss though AKT1. In line with this, the nitric oxide (NO) that accumulates under salinity stress also inhibits the K^+^ uptake mediated by AKT1. The link is indirect since NO triggered the accumulation of pyridoxal 5′-phosphate (PLP), an active form of vitamin B6, that in turn repressed the activity of AKT1 in *Xenopus* oocytes and *Arabidopsis* root protoplasts (Xia et al., 2014).

In *Arabidopsis*, the voltage-gated channel GORK (guard cell outward-rectifying K ^+^) is the major outward-rectifying K^+^ channel in guard cells where it contributes to K^+^ efflux for decreasing turgor and stomatal closure (Ache et al., 2000; Hosy et al., 2003). In addition, GORK is expressed in root outer cell layers (epidermal, root hairs, and cortex) of *Arabidopsis* and thus GORK is considered a major pathway for stress-induced K^+^ leakage from root cells, e.g. by exposure of roots to high salt (Ivashikina et al., 2001; Demidchik et al., 2010; Demidchik et al., 2014). Production of hydroxyl radicals (HO˙) in salinized roots stimulates a dramatic K^+^ efflux mediated by GORK from root cells (Demidchik et al., 2010). The oxidative and salt stresses cause programmed cell death (PCD) and collapse membrane potential in root cells of *Arabidopsis thaliana* in a K^+^-dependent manner. Accordingly, the *Arabidopsis gork1-1* mutant showed no K^+^ outwardly directed currents in response to HO˙. Besides, after exposure to high NaCl levels, the mutant *gork1-1* displayed lower activity of proteases and endonucleases for PCD, which in the wild type was dramatically enhanced by K^+^ loss in root cells (Demidchik et al., 2010).

Both the expression level and channel activity of GORK are significantly upregulated by increasing levels of the abscisic acid (ABA) and jasmonate. Stimuli that elevated endogenous ABA concentrations, e.g. drought, osmotic stress, or cold, led to the up-regulation of *GORK* transcripts (Becker et al., 2003; Suhita, 2004) while treatment with salicylic acid inhibited the presence of active GORK channels and improved salinity tolerance through prevention of K^+^ efflux. Recent studies demonstrated that calcium-dependent protein kinase 21 (CPK21) phosphorylated GORK and suggested that 14-3-3 proteins control GORK activity through binding with CPK21. This kinase phosphorylates three amino acid residues in the C-terminus of GORK, T344, S518, and S649. Binding of 14-3-3 to CPK21 strongly stimulated its kinase activity and increased GORK phosphorylation (van Kleeff et al., 2018). On the other hand, the phosphatase AtPP2CA interacts physically with GORK inhibiting its current (Lefoulon et al., 2016). Thus, AtPP2CA could have an antagonist role to CPK21 on the regulation of GORK (van Kleeff et al., 2018). These results imply that the salinity-induced membrane depolarization together with the Ca^2+^- and CPK21-dependent phosphorylation act together to activate GORK and to repolarize the plasma membrane by means of releasing part of the cytosolic K^+^. Moreover, the peak of the salt-induced K^+^-efflux in the *aha2* mutant, devoid of a major isoform of the plasma membrane H^+^-ATPase, was stronger and more sustained than in the wild-type, suggesting that H^+^-pumps take over membrane repolarization after the initial K^+^-loss to re-enact K^+^ uptake (van Kleeff et al., 2018). Recently, Saponaro et al. (2017) showed that 14-3-3 proteins are also capable of modulating KAT1, although in this case 14-3-3 bound directly to the KAT1 C-terminus affecting both the voltage dependency of the channel and the number of channel molecules in the membrane (Sottocornola et al., 2008).

K^+^-H^+^ symport has long been considered the likely catalytic mechanism of plant KT/HAK/KUP transporters based on the demonstration that K^+^-H^+^ symport operates in K^+^-starved *Neurospora crassa* and on thermodynamical considerations regarding the steep K^+^ gradient that KT/HAK/KUP proteins are able to achieve across cell membranes that exceeds what could be reached by coupling the K^+^ uptake to the membrane potential solely (Rodriguez-Navarro, 2000). Until recently, efforts to express plant KT/HAK/KUP proteins in *Xenopus* oocytes to measure K^+^ currents had failed, but work with the DmHAK5 transporter from Venus flytraps showed that co-expression of the corresponding cRNA with that of CBL9/CIPK23 (but not DmHAK5 alone) generated inward K^+^ and Rb^+^ currents in *Xenopus* oocytes that were stimulated by low external pH (Scherzer et al., 2015). Moreover, salt bladders of the halophyte *Chenopodium quinoa* that accumulate salts to very high concentrations express a HAK-like activity driving high-affinity and selective K^+^ uptake that was dependent on acidic external pH and by the CIPK23/CBL1 kinase module of *Arabidopsis* (Bohm et al., 2018). Electrophysiological recordings in rice roots showed that the activity of OsHAK1 was strongly electrogenic and depolarizing. Plots of the OsHAK1-dependent K^+^-induced membrane depolarization had a slope of 29 mV per decade of external K^+^ concentration, suggesting the co-transport of two monovalent cations (a 59 mV slope is to be expected from an uniprot transport moving only single K^+^ ions) (Nieves-Cordones et al., 2017). Together, these data strongly suggest that plant KT/HAK/KUP proteins operate as K^+^-H^+^ symporters. Residues involved in K^+^ binding and/or transport have not been identified; however, mutant proteins with residue substitutions of members of the KT/HAK/KUP family have been described as showing modified affinity for K^+^, Na^+^, and/or Cs^+^, or increased Vmax (Aleman et al., 2014).

HAK1-like transporters are subject to complex transcriptional and post-translational regulations, although studies have been carried out almost exclusively in *Arabidopsis* AtHAK5 (Jung et al., 2009; Rubio et al., 2014; Ragel et al., 2015). Under any stress conditions that directly affect K^+^ acquisition, such as K^+^ deprivation or salinity, high-affinity K^+^ uptake systems should be transcriptionally or post-translationally activated in order to maintain the K^+^ supply and K^+^/Na^+^ homeostasis. Accordingly, all characterized HAK1-like transporters exhibit low expression levels in roots under control conditions, are highly up-regulated upon K^+^ deprivation and rapidly down-regulated when K^+^ is resupplied (reviewed by (Li et al., 2018)). Furthermore, it has been commonly observed that other ions, particularly NH_4_^+^, NO_3_^−^, Na^+^, and Pi, also regulate the expression of *HAK1*-like genes and not always in the same way (Nieves-Cordones et al., 2019). For example, NH_4_^+^ reduces the transcriptional induction by K^+^ starvation of the pepper *CaHAK1* (Martinez-Cordero et al., 2005) and *Arabidopsis AtHAK5* (Qi et al., 2008), but enhances the expression of *LeHAK5* in tomato (Nieves-Cordones et al., 2007). The presence of NaCl prevents the induction of *LeHAK5* by K^+^ starvation (Nieves-Cordones et al., 2007), but provokes a strong and transient up-regulation of *HvHAK1* (Fulgenzi et al., 2008). Thus, the *Arabidopsis* model cannot be completely extended to other plant species, crops among them. In contrast to HAK1-like transporters, KT/HAK/KUP proteins belonging to clusters II–V show diverse expression patterns and most of them do not exhibit transcriptional regulation in response to K^+^ deficiency (Ahn et al., 2004; Li et al., 2018). For example, *AtKUP7* (cluster V, plasma membrane) transcript is not induced by low-K^+^ (Han et al., 2016) and *AtKUP12* (cluster III, chloroplast) is down-regulated after K^+^ resupply (Armengaud et al., 2004).

Regarding the transcriptional regulation of genes encoding HAK1-like transporters, it has been shown that the effect of the nutrient deficiency and salt stresses on transcriptional expression of *AtHAK5* and *LeHAK5* is associated with changes in the root cell membrane potentials (Nieves-Cordones et al., 2008; Rubio et al., 2014); the hyperpolarization of the plasma membrane of root cells induces transcription of both genes. Supporting this, *ThHAK5* of *Thellungiella halophila* (salt cress, a.k.a. *Eutrema salsuginea*) is expressed to higher levels than *AtHAK5* under salt stress, while roots of *T. halophila* maintained a more negative membrane potential than *Arabidopsis* roots (Volkov and Amtmann, 2006; Alemán et al., 2009b; Rubio et al., 2014). Besides membrane hyperpolarization, the expression of *AtHAK5* is also induced, under K^+^-limiting conditions, as result of signaling cascades that involve ROS production, phytohormones, and transcription factors. Low-K^+^ stress, alike other nutrient-deprived conditions, promotes an increase of ethylene that positively regulates ROS production in roots (Shin and Schachtman, 2004; Jung et al., 2009). Roots deprived of K^+^ induce the expression of genes involved in ethylene biosynthesis and signaling, and in ROS metabolism, promoting two-fold higher levels of ethylene and the increase in hydrogen peroxide (H_2_O_2_) concentrations. Both ethylene and ROS give rise to enhanced transcription of *HAK5* in *Arabidopsis* and tomato (Rodenas et al., 2018). In *Arabidopsis*, H_2_O_2_ produced by the NADPH oxidase RHD2/RbohC regulates the expression of *AtHAK5* in response to K^+^ deficiency (Shin and Schachtman, 2004) and it has been proposed that peroxidase RCI3 (Rare Cold Inducible gene 3) contributes to ROS production during *Arabidopsis* root response to K^+^ deficiency (Kim et al., 2010). In the case of ethylene-induced *AtHAK5* transcription, the intermediaries in ethylene signaling CTR1 (Constitutive Triple Response (1) and EIN2 (Ethylene Insensitive (2) are partially involved. Results also suggest the existence of other signaling pathways or an EIN2-independent ethylene route that may play an important role in low-K^+^ signaling (Jung et al., 2009). Genetic hierarchy indicates that ethylene signaling acts upstream of ROS when plants are deprived of K^+^ (Jung et al., 2009). Nevertheless, it has also been speculated that a positive feedback may stimulate ethylene-induced ROS production (Wang et al., 2002). Other hormones have been shown to be involved in K^+^ deprivation signaling and response, for instance jasmonic acid (Armengaud et al., 2004), auxin (Jung et al., 2009; Hong et al., 2013), ABA (Kim et al., 2010), cytokinins (Nam et al., 2012), and gibberellins through DELLA proteins (Oliferuk et al., 2017). Cytokinins are known to regulate macronutrient homeostasis by controlling the expression of nitrate, phosphate, and sulfate transporters. Cytokinin content decreases under K^+^-starved conditions, and cytokinin-deficient mutants, under same conditions, display enhanced accumulation of both ROS and *AtHAK5* transcripts (Nam et al., 2012). By contrast, cytokinin-receptor mutants lost the responsiveness to low-K^+^, including ROS accumulation and root hair growth. Interestingly, the cytokinin/ethylene ratio is positively correlated with tomato shoot biomass, suggesting that the balance between both hormones is important in determining the plant vigor at low-K^+^ supply, but with an inverse role in tomato compared to *Arabidopsis*, where cytokinin/ethylene ratio was negatively correlated with tolerance to K^+^ deprivation (Jung et al., 2009; Nam et al., 2012).

In addition to low nutrient conditions, salt stress (and presumably other abiotic stresses) results in modifications of *AtHAK5* expression or the low-K^+^ response. Mild salt stress does not induce *AtHAK5* expression but its expression levels gradually increased following an increase in NaCl concentrations (Ahn et al., 2004; Hong et al., 2013). This suggests that plants may recognize high Na^+^ levels as K^+^ deprivation. However, the induction of *AtHAK5* expression by low K^+^ was suppressed by salt stress in *Arabidopsis* (Nieves-Cordones et al., 2010), but not in *T. halophila* (Alemán et al., 2009a). As discussed above, under salt stress conditions, *T. halophila* registers a more negative root membrane potential than *A. thaliana* (Volkov and Amtmann, 2006), which may explain the expression of *ThHAK5* under these conditions (Alemán et al., 2009a).

In recent years, several transcription factors (TFs), and their target sequences, have been identified in the *AtHAK5* promoter. Among them, ARF2 (Auxin Response Factor 2) is the only one described so far to work as negative regulator of *AtHAK5* transcription (Zhao et al., 2016). Interestingly, ARF2 has been found to be involved in many phytohormone-signaling pathways, but it seems not to participate in auxin signaling. Under K^+^-sufficient conditions, channel-mediated K^+^ uptake would be energetically more favorable than symport through AtHAK5, and hence *AtHAK5* should be shut down (Zhao et al., 2016). In those conditions, ARF2 binds to the auxin-responsive elements (AuxREs) within the *AtHAK5* promoter and represses transcription. When plants are subjected to low-K^+^ stress, ARF2 is rapidly phosphorylated by an unknown kinase and loses DNA binding activity. ARF2 is removed from the *AtHAK5* promoter, which relieves the repression on *AtHAK5* transcription. In turn, other TFs bind to the *AtHAK5* promoter and activate its transcription. These TFs up-regulating *AtHAK5* expression under K^+^ starvation include RAP2.11, which binds to the ethylene-responsive element (ERE) and the GCC-box of the *AtHAK5* promoter, and whose expression is stimulated by ethylene and ROS, alike *AtHAK5* (Kim et al., 2012). TFs DDF2, JLO, bHLH121, and TFII_A also interact with the upstream region of *AtHAK5*, but the specific binding motif for each of them has not been identified yet (Hong et al., 2013). All of these transcription factors are sufficient to activate *AtHAK5* expression in heterologous systems, but none of them is absolutely required. When K^+^ is resupplied, ARF2 becomes dephosphorylated again and represses *AtHAK5* expression (Zhao et al., 2016). Thus, it is apparent that regulation of the activity of TFs acting on *AtHAK5* transcription (positively or negatively) is necessary to determine cooperatively the accumulation of the corresponding transcripts (Santa-Maria et al., 2018).

**Figure 3:**
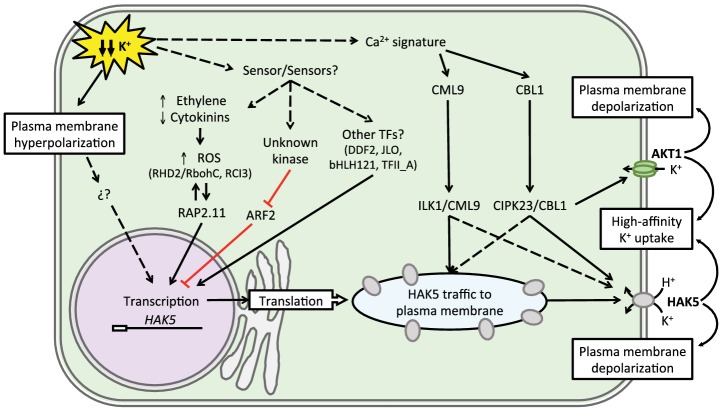
Regulatory circuitry regulating AtHAK5 expression and activity. K^+^ starvation is probably sensed as hyperpolarization of the plasma membrane, which, together with elevated ethylene and ROS levels, leads to expression of the *HAK5* gene. Several transcriptional activator and repressor factors have been identified, but their placement in specific signaling pathways is uncertain. Subsequently, calmodulin-like (CML) and calcineurin B-like (CBL) Ca^2+^-binding proteins recruit and activate protein kinases ILK1 and CIPK23 that facilitate the trafficking of HAK5 to the plasma membrane and its biochemical activation, respectively. CIPK23 also stimulates the K^+^-uptake channel AKT1. K^+^ replenishment depolarizes the membrane and returns the system to homeostatic levels.

Although a general nutrient deprivation stimulus is sufficient for the transcriptional activation of *AtHAK5* and *LeHAK5* genes, a reduction of internal K^+^ is required for the induction of a functional HAK5-mediated high-affinity K^+^ uptake in *Arabidopsis* and tomato roots (Rubio et al., 2014), suggesting the existence of post-transcriptional regulation *in planta*. Recently, it was shown that activation of high-affinity K^+^ uptake mediated by AtHAK5 (Ragel et al., 2015), DmHAK5 from Venus flytraps (Scherzer et al., 2015), and CqHAK from quinoa (Bohm et al., 2018) is mediated by the CBL-interacting protein kinase (CIPK)/calcineurin B-like protein (CBL) complex comprising CIPK23 and CBL1/9 proteins of *Arabidopsis*. Notably, this CIPK23/CBL1,9 module also activates AKT1 channel, that together with AtHAK5 constitutes the main K^+^ uptake pathway in *Arabidopsis* roots (Xu et al., 2006; Lee et al., 2007). Both the protein kinase AtCIPK23 and the Ca^2+^ sensor AtCBL1 are necessary and sufficient for activation of the high-affinity K^+^ transporter AtHAK5 in yeast (Ragel et al., 2015). Besides AtCBL1, other CBLs (AtCBL8/9/10) are able to bind AtCIPK23 and activate AtHAK5 to complement K-uptake defective yeast growth. The reduction in the K^+^ concentration produces a specific Ca^2+^ signature in the cytosol (Figure 3) (Behera et al., 2017) that would be recorded by AtCBL1, promoting CIPK23/CBL1 complex formation, and the activation of AtHAK5 by phosphorylation at the cytosolic N-terminus (Ragel et al., 2015), in a similar way that was described for AKT1 (Xu et al., 2006; Lee et al., 2007). The enhancement of growth at low-K^+^ of yeast cells co-expressing AtHAK5, AtCIPK23, and AtCBL1 seems to result from modification of the kinetic properties of the transporter (Km decrease and Vmax increase), likely through the phosphorylation-induced conformational changes of AtHAK5 (Ragel et al., 2015). However, since physical interaction between CIPK23/CBL1 and AtHAK5 is also required for full activation of AtHAK5 in yeast, the trafficking of the transporter to plasma membrane has been proposed as a second mechanism of AtHAK5 regulation by CIPK23/CBL1 complex. Supporting this idea, the AtHAK5 protein was mainly detected in the endoplasmic reticulum of K^+^-sufficient plants, while K^+^ starvation produced an enrichment of AtHAK5 protein in the plasma membrane (Qi et al., 2008). In heterologous systems, the *Arabidopsis* CIPK23/CBL1,9 complex enabled the activation of various members from clade I of KT/HAK/KUP transporters, such as pepper CaHAK1 (Ragel et al., 2015) and Venus flytrap DmHAK5 (Scherzer et al., 2015), but not of tomato SlHAK5 or the *Eutrema salsuginea* EsHAK5 (Ragel et al., 2015). These results suggested that the activation mechanism by CIPK23/CBLs complexes is evolutionarily conserved, but not the phosphorylation site and/or the target sequence recognition, which may vary among distant plant species. Accordingly, a quimeric tomato HAK5 protein that contained the 15 first amino acids of CaHAK1 could be activated by CIPK23/CBL1 in yeast (Ragel et al., 2015).

Since the two main contributors to K^+^ uptake in *Arabidopsis*, AKT1 and HAK5, are regulated by the CIPK23/CBL1,9 complex, the coordinated regulation of these transport systems deserves attention. Under K^+^-sufficient conditions, K^+^ uptake by HAK5 would be energetically more expensive than permeation through the AKT1 channel. The H^+^-pumping activity of plasma membrane ATPases, which is used for many secondary transport processes, creates *per se* an electrical charge (negative inside) that suffices to draw significant amounts of K^+^ into the cytosol. At a regular steady membrane potential of −120 to −180 mV, root epidermal cells could sustain a 100–1,000-fold inward-directed gradient of K^+^. However, coupling K^+^ uptake to H^+^ influx not only returns H^+^ to the cytoplasm but also is more depolarizing than simple K^+^ permeation, which in turn imposes a greater demand on the H^+^-pumps and ATP consumption. We speculate that under such conditions of K^+^ sufficiency, the plasma membrane is not hyperpolarized (or not enough), signaling phytohormones are not produced, and therefore transcription of *HAK5* is not activated. AKT1 would be operational through the physical interaction with CIPK23 (and possibly other CIPKs) (Lee et al., 2007). As the K^+^ concentration outside decreases, the function of AKT1 becomes increasingly hampered and full activation by the Ca^2+^-dependent CIPK23/CBL1,9 complex is required to sustain K^+^ uptake, while the KC1 safeguard prevents K^+^ leakage through AKT1 (Wang et al., 2016). CIPK23 is known to display different states of activation, depending on factors that affect the activation of CIPKs by upstream kinases (Barajas-Lopez et al., 2018) and CBL binding (Chaves-Sanjuan et al., 2014). Thus, a mild K^+^ deprivation may produce a partially activated CIPK23 that would be competent for activating AKT1 but not HAK5, whereas severe K^+^ deprivation leads to *HAK5* transcription and to full activation of CIPK23, which would then be competent for activating HAK5 (Ragel et al., 2015). The *CIPK23* is itself induced transcriptionally by low-K^+^ stress (Xu et al., 2006), which could also enhance the response to nutritional stress.

The participation of Ca^2+^ sensors in high-affinity K^+^ uptake could mechanistically connect K^+^ starvation with other abiotic stresses, for instance: salinity, water availability, oxygen deficiency (hypoxia) or absence (anoxia), mechanical stress, cold stress, heavy metal stress, and other nutrient deprivations, all sharing cytosolic free Ca^2+^ as a second messenger (Wilkins et al., 2016). Current thinking is that the specificity of Ca^2+^ signaling is determined by the amplitude and duration (and possible oscillation) of the cytosolic Ca^2+^ increase, often referred to as the “calcium signature” that is elicited by the stimulus. K^+^ deficiency evokes two successive Ca^2+^ signals in roots exhibiting different spatial and temporal specificity (Behera et al., 2017). The first one is characterized by a transient and fast Ca^2+^ increase within 1 min in the postmeristematic elongation zone (most prominently in the vascular tissue and endodermis), followed by a Ca^2+^ return nearly to basal concentrations in <7 min. The second wave (secondary Ca^2+^ response) occurs after several hours (18 h) as sustained Ca^2+^ elevation in defined tissues of the elongation and root hair differentiation zones. It has been proposed that this secondary Ca^2+^ elevation would contribute to long-term adaptation responses (Behera et al., 2017). These differential Ca^2+^ signatures could also underpin the step-wise activation of CIPK23/CBL1,9 discussed above. Ca^2+^-sensing proteins with diverse Ca^2+^ affinities, subcellular localizations, and downstream target specificities may perceive Ca^2+^ signatures differentially and transduce them into adequate downstream signaling responses (Hashimoto and Kudla, 2011).

Besides the CIPK23/CBL1,9 module, evidence for a second Ca^2+^-dependent pathway acting on AtHAK5 during low-K^+^ signaling has been recently provided (Brauer et al., 2016). The INTEGRIN LINKED KINASE1 (ILK1) interacts with AtHAK5 and promotes its accumulation, likely by phosphorylation of AtHAK5, albeit this could not be proved *in vitro*. ILK1 activity contributes to growth during extreme K^+^ limitation where AtHAK5 is the only transport system contributing to K^+^ uptake. ILK1 interacts with the calmodulin-like protein 9 (CML9), which invokes Ca^2+^ signaling, and both proteins were needed to promote AtHAK5 accumulation in the membrane fraction of *Nicotiana benthamiana* leaves, leading to the suggestion that ILK1 and CML9 promote HAK5 maturation and transport from the endoplasmic reticulum to the plasma membrane. It remains to be investigated whether ILK1 functions in parallel or in coordination with the CIPK23/CBL pathway to modify AtHAK5 activity.

In addition to the interaction with kinases, the modulation of KT/HAK/KUP transporters through physical interactions among different subunits (homomers or heteromers formation) has been suggested. Physical self-interaction among subunits of AtKUP4 has been described (Daras et al., 2015). In the event that KT/HAK/KUP proteins formed a tetrameric structure similar to that of *Shaker* channels, the conserved sequence GVVYGDLGTSPLY in the first TM fragment of each subunit would line the pore, where the three conserved glycine residues of each subunit may operate as a selectivity filter (Rodriguez-Navarro, 2000).

In summary, the integration of post-translational regulation, including a specific Ca^2+^ signature, protein interactions, phosphorylation events, and reallocation of transporters to the plasma membrane, as well as the transcriptional regulation governed by plasma membrane hyperpolarization, ROS production, and hormonal response pathways, all of them integrate in the regulation of HAK1-like K^+^ uptake systems in plant cells under different K^+^ starvation conditions. Figure 3 summarizes the transcriptional and post-translational regulation of AtHAK5.

The *Arabidopsis* mutant lacking HAK5 and AKT1 still takes up K^+^ and shows residual growth at external K^+^ concentration above 1 mM, indicating the existence of additional compensatory transport system(s) (Pyo et al., 2010; Rubio et al., 2010). Because this low-affinity uptake is largely sensitive to Ca^2+^ and other divalent metals, permeates Cs^+^, and is inhibited by cyclic nucleotides, non-selective cyclic nucleotide-gated cation channels (CNGC) are likely candidates for this alternative system (Caballero et al., 2012). Indeed, genetic data have implicated AtCNGC3 and AtCNGC10 in K^+^ uptake because knock-out and knock-down lines have reduced K^+^ contents (Kaplan et al., 2007). Moreover, AtCHX13, a plasma membrane cation/proton antiporter up-regulated by K^+^ starvation, has been involved in root K^+^ uptake (Zhao et al., 2008). AtKUP7 is preferentially expressed in *Arabidopsis* roots, and may also be instrumental in K^+^ uptake and in K^+^ loading into xylem sap, affecting K^+^ translocation from roots to shoots (Han et al., 2016). In rice, OsHAK1 and OsAKT1 fulfill the same functions than the *Arabidopsis* counterparts HAK5 and AKT1 (Li et al., 2014; Chen et al., 2015; Nieves-Cordones et al., 2017). In addition, OsHAK5 has been related to high-affinity K^+^ uptake and in the release of K^+^ into the xylem (Nieves-Cordones et al., 2016b). Table 2 summarizes the range of K^+^ concentrations in soil at which each AKT1-like channel and HAK1-like transporter contributes to high-affinity K^+^ uptake in *Arabidopsis* and rice (Aleman et al., 2011; Nieves-Cordones et al., 2016a).

Of note is that HKT2-like transporters have been implicated in K^+^ nutrition in cereals. The rice protein OsHKT2;1 provides a major pathway for root high-affinity Na^+^ uptake that supports plant growth under limiting K^+^ supply (Horie et al., 2007; Haro et al., 2010). Under K^+^ starvation, Na^+^ can partially compensate for K^+^ as osmoticum and change balance (Rodriguez-Navarro and Rubio, 2006; Alvarez-Aragon and Rodriguez-Navarro, 2017), and indeed high-affinity Na^+^ uptake has been observed in roots of several species (Haro et al., 2010). Rates of Rb^+^ influx between WT and *hkt2;1* roots did not reveal significant differences, but *hkt2;1* had lower Na^+^ contents (Horie et al., 2007). Thus, the possible involvement of OsHKT2;1 in root K^+^ uptake could not be verified. Although the activity of HKTs may influence the K^+^ status of plants, particularly under saline stress, HKTs appear to be determinants for salt tolerance with no significant role in K^+^ nutrition (Hamamoto et al., 2015).

## Compartmentation and Storage into Vacuoles

Once K^+^ is inside the root symplast, it may be stored in vacuoles locally, or transported to the shoot *via* xylem and accumulated in aerial tissues. Plants accumulate large amounts of K^+^ in their vacuoles, surpassing purely nutritional requirements. In K^+^-sufficient plants, K^+^ content can reach up to 10% of plant dry weight, thereby exceeding the ca. 2% that supports near-maximal growth rates (White and Karley, 2010). The vacuolar K^+^ pool plays a chief biophysical function, i.e. the lowering of osmotic potential to draw water, generate turgor, and drive cell expansion. Because vacuoles occupy most of the intracellular volume of plant cells and are the main cellular reservoir for K^+^, changes in tissue K^+^ concentration are largely a reflection of the dynamics of the vacuolar pool. Cytosolic K^+^ concentration will decline below the optimal set point of 80–100 mM only when the vacuolar K^+^ reserve has been depleted below the thermodynamic equilibrium with the cytosolic pool (Walker et al., 1996). Conversely, surplus K^+^ is placed into the vacuole to maintain cytosolic K^+^ within narrow limits independently of K^+^ abundance in the growth medium.

In contrast to the plasma membrane, accumulation of K^+^ in the vacuole depends on the coordinated activity of tonoplast H^+^ pumps and secondary K^+^ transporters that link K^+^ fluxes to the dissipation of the pH gradient or the electrical membrane potential created by the asymmetric distribution of charges. The vacuolar H^+^-ATPase (V-ATPase) and the pyrophosphatase of the tonoplast (PPase) pump H^+^ toward the vacuolar lumen and generate pH gradients of 1–2 pH units (acidic inside) and an electrical charge (membrane potential) of 20–40 mV that is positive in the vacuolar lumen relative to the cytosol. This means that positively charged K^+^ ions are excluded from K^+^-replete vacuoles unless transport is coupled to an energy-dependent uptake mechanism, whereas efflux is driven by vacuolar channels permeating K^+^ downhill its electrochemical gradient. A K^+^/H^+^ antiporter energized by the pH gradient across the tonoplast was long suggested to catalyze vacuolar K^+^ accumulation (Walker et al., 1996; Carden et al., 2003), but the molecular identity of the underlying transporter(s) has remained elusive until recently. Vacuolar NHX-type exchangers have been shown to serve this critical function in plant cells (Venema et al., 2002; Venema et al., 2003; Leidi et al., 2010; Bassil et al., 2011; Barragan et al., 2012).

NHX exchangers were originally described as Na^+^/H^+^ antiporters able to confer salt tolerance by driving the sequestration of excess Na^+^ into vacuoles (Blumwald, 2000). However, the underlying mechanism remained uncertain because the salt tolerance of transgenics overexpressing NHX proteins from various sources did not always correlate with enhanced Na^+^ accumulation (Jiang et al., 2010). Moreover, biochemical studies established that NHX proteins catalyze Na^+^/H^+^ and K^+^/H^+^ exchange with similar affinities (Venema et al., 2002). The recent meta-analysis of a large number of publications reporting tolerance phenotypes imparted by exchangers of the Cation/Proton Antiporter Family 1 (CPA1, which includes NHX proteins) concluded that the effect on K^+^ status was generally more pronounced than on Na^+^ content (Ma et al., 2017). An informative work showed that overexpression of the AtNHX1 in tomato induced K^+^-deficiency symptoms despite transgenic plants having greater K^+^ contents than controls (Leidi et al., 2010). The intense sequestration of K^+^ in NHX1-overexpressing plants reduced cytosolic K^+^ activity, primed the induction of the high-affinity K^+^ uptake system, and elicited an array of metabolic and hormonal disorders related to K^+^ deprivation (Leidi et al., 2010; De Luca et al., 2018). Notwithstanding these unintended effects resulting from NHX overexpression, NHX proteins do increase salt tolerance, presumably because retention of cellular K^+^ is a requisite for adaptation to a saline environment (Jiang et al., 2010). Salinity stress elicits depolarization of the root plasma membrane and ROS production, both of which open outward-rectifying K^+^ channels that discharge K^+^ to re-build the membrane potential (Shabala and Pottosin, 2014). The salinity-induced K^+^ loss implies the need to replenish the cytosolic K^+^ pool by withdrawing K^+^ stored in vacuoles (Cuin et al., 2003; Leidi et al., 2010).

Deletion of *NHX1* and *NHX2* genes encoding the two major vacuolar NHX isoforms resulted in the inability to compartmentalize K^+^ and, surprisingly, in sensitivity to K^+^ supply at concentrations that did not compromise the growth of control plants (Bassil et al., 2011; Barragan et al., 2012). Moreover, *nhx1 nhx2* mutant lines showed dysfunctional stomatal activity, with impaired opening and closure (Barragan et al., 2012; Andrés et al., 2014). The rapid uptake and release of K^+^ and anionic organic acids by guard cells, mostly in the vacuolar compartment, drives the movements of stomata. Changes in the volume and shape of guard cells run in parallel with intense remodeling of vacuoles (Gao et al., 2005; Tanaka et al., 2007). Disruption of K^+^ accumulation in the guard cells of *nhx1 nhx2* mutant plants correlated with more acidic vacuoles and the disappearance of the highly dynamic remodeling of vacuolar structure associated with stomatal movements (Andrés et al., 2014).

Electrophysiological recordings of channel activities in the tonoplast have identified fast vacuolar (FV), slow vacuolar (SV), and K^+^-selective vacuolar (VK) cation channels that mediate the release of vacuolar K^+^ (Hedrich, 2012). The VK currents have been assigned to two-pore K^+^ (TPK) channels (Gobert et al., 2007). TPK1, 2, 3, and 5 of *Arabidopsis* are located in the tonoplast, while TPK4 is in plasma membrane. TPK1 currents are independent of the membrane voltage but sensitive to cytosolic Ca^2+^ and regulated by calcium-dependent protein kinases (CDPKs) and 14-3-3 protein binding (Latz et al., 2013). Also in *Arabidopsis*, the TPC1 channel accounts for the SV current (Peiter et al., 2005). TPC1 is voltage-dependent and non-selective, allowing K^+^ and Na^+^ to permeate toward the cytosol. Whether TPC1 also permeates Ca^2+^ or Ca^2+^ is only an effector of TPC1 gating is a matter of controversy (Hedrich et al., 2018). TPC channels are activated by a decrease in transmembrane potential and increased cytosolic Ca^2+^, and inhibited by low luminal pH and Ca^2+^. Structurally, TPC1 resembles two subunits of voltage-dependent *Shaker*-like channel fused in tandem, and two cytosolic EF hands in between (Guo et al., 2016; Kintzer and Stroud, 2016). The ubiquitous nature of TPC channels and the magnitude of the SV/TPC currents are such that TPC channels are capable of contributing substantially to cellular K^+^ homeostasis. Accordingly, the transcriptome of the *tpc1* loss-of-function mutant of *Arabidopsis* is reminiscent of profiles that were obtained under K^+^ limitation (Bonaventure et al., 2007b). However, plants lacking TPC1 function are not impaired in growth and development. This may indicate that the TPC1 channel is closed most of the time and opens upon specific inputs or under stress. Current thinking is that TPC1 is part of a Ca^2+^/ROS relay that propagates stress signals (Choi et al., 2014; Evans et al., 2016). The gain-of-function mutant *fou2* results in a hyperactive TPC1 channel with an altered voltage-dependent gating behavior that increases the probability of the channel to be open under physiological vacuolar potentials. As a consequence of this “leaky” channel, the *fou2* mutant plant behaves as being wounded and shows elevated levels of the stress hormone jasmonate (Bonaventure et al., 2007a).

Several KT/HAK/KUP transporters have been localized to the tonoplast (Table 1). They are thought to force the energetically uphill release of K^+^ into the cytoplasm under chronic K^+^ starvation, in which the cytosolic concentration of K^+^ could be low enough to impair the discharge of luminal K^+^ by tonoplast channels (Ahmad and Maathuis, 2014).

## Long-Distance Transport and Inter-Organ K^+^ Partitioning

Potassium absorbed by peripheral root cells and not compartmentalized in vacuoles must be transported to the upper parts of the plant through the xylem (Gaymard et al., 1998; Park et al., 2008; Ahmad and Maathuis, 2014). This step is critical in the long-distance distribution of K^+^ from roots to the upper parts of the plant, and is driven by negative pressure (pulling) created by evaporation of water from leaves. The osmotic water uptake that is caused by nutrient absorption in the root also provides a positive force, known as root pressure, from roots to xylem vessels. Under regular K^+^ supply, symplastic K^+^ diffusion to the xylem through the stele may contribute sufficiently to K^+^ transport from root to shoot (Yang et al., 2014). Moreover, K^+^ is highly mobile within plants, exhibiting cycling between roots and shoots *via* xylem and phloem (Ahmad and Maathuis, 2014). In this section, we will review K^+^ channels, KT/HAK/KUP carriers, and HKT transporters that are involved in long-distance transport of K^+^ in plants.

Potassium channels SKOR and AKT2 play an important role in K^+^ translocation *via* xylem and phloem. SKOR (Stelar K^+^ Outward Rectifier) is expressed in root stele cells (pericycle and xylem parenchyma cells) of *Arabidopsis*, where it mediates K^+^ secretion by xylem parenchyma cells of roots and toward the xylem vessels (Gaymard et al., 1998). SKOR, being an outward-rectifying channel, opens upon membrane depolarization to allow cytosolic K^+^ efflux. In addition, the gating of SKOR is sensitive to extracellular K^+^ concentration, with a maximum activity around 10 mM K^+^. In the presence of ample external K^+^, the channel opens at less negative membrane voltages, thereby minimizing the risk to serve as an undesirable K^+^-influx pathway. This behavior of SKOR is achieved by a complex interplay between the pore region and the “S6 gating domain” localized in the last transmembrane segment, which contains three amino acid residues, D312-M313-I314, that acquire great relevance in coupling K^+^ sensitivity and gating of the channel (Johansson et al., 2006). At high external K^+^ concentration, the pore region is more rigid and strongly interacts with the S6 gating domain stabilizing the channel in a closed state. On the other hand, with a low external K^+^ concentration, the pore region is less occupied, more flexible, and does not interact with the surrounding transmembrane domains anymore.

The expression of *SKOR* is inhibited by abscisic acid (ABA) synthesized during water stress. This suggests that diminished K^+^ transport to the xylem in response to ABA allows osmotic adjustment of roots to the drying soil (Gaymard et al., 1998). Intra- and extra-cellular acidification also induced a decrease of SKOR currents at the macroscopic and single-channel levels without affecting macroscopic gating parameters and the single channel conductance. This decrease of SKOR currents could be due to a reduction in the number of channels available for activation, which could help preventing K^+^ loss from roots toward the shoot tissue (Lacombe et al., 2000a). Hydrogen peroxide exhibits the opposite effect on SKOR. Treatment with H_2_O_2_ increases SKOR outward currents and decreases its half activation time. Analyses in heterologous expression systems showed that SKOR sensibility to ROS is a voltage-dependent process produced by oxidation of Cys168 located on the S3 α-helix within channel (Garcia-Mata et al., 2010). Thus, upon acute depolarization of plasma membrane induced by salinity, SKOR in xylem parenchyma cells can be rapidly activated to mediate K^+^ loading into the xylem. After the plasma membrane potential is restored by increased H^+^-ATPase activity, SKOR-dependent K^+^ release from root stelar cells to the xylem by membrane depolarization is suppressed. Then, accumulated ROS under salinity could, in turn, activate SKOR channels to allow xylem K^+^ loading. This may require a highly coordinated mechanism to ensure efficient xylem K^+^ loading in salt-stressed plants.

Large quantities of K^+^ recirculate from roots to shoots *via* the xylem and subsequently return to the roots *via* the phloem (Thompson and Zwieniecki, 2005; De Schepper et al., 2013). The magnitude of the K^+^ flux recirculated from the shoots to the roots would constitute a signal by which the growing shoots could communicate to roots their K^+^ requirement and regulate K^+^ secretion into the xylem sap (and eventually root K^+^ uptake). *AKT2* is mainly expressed in the phloem both in leaves and roots (Deeken et al., 2000; Lacombe et al., 2000b), where the AKT2 channel protein plays a dual role by loading K^+^ in source tissues and unloading K^+^ in sink organs (Gajdanowicz et al., 2011). AKT2 is the only weak inward-rectifier characterized in *Arabidopsis* (Dreyer et al., 2001; Cherel et al., 2014). The protein phosphatase PP2CA interacts with AKT2 to induce both the inhibition of the channel current and the enhancement of its inward rectification (Cherel et al., 2002). When expressed in mammalian COS cells and *Xenopus* oocytes, AKT2 channel exhibited two gating modes that were dependent on phosphorylation by endogenous cAMP-dependent protein kinase A (PKA) (Michard et al., 2005a,b). In mode 1, the non-phosphorylated channel behaves as a weak inward-rectifier. In mode 2, phosphorylated AKT2 is permanently open and able to conduct K^+^ in the inward and the outward directions. Two serine residues, S210 and S329, located in the pore inner mouth that likely undergoes conformational changes on voltage-dependent movements, were identified as targets for phosphorylation (Michard et al., 2005a). Nonetheless, it was proposed that post-translational modifications in these positions alone are not enough to completely convert AKT2 from an inward-rectifying to a non-rectifying channel. A lysine within the voltage sensor enables AKT2 to sense its phosphorylation status and to change between the two modes. Replacement of the lysine by serine or arginine displays an AKT2 inward-rectifier (Michard et al., 2005a; Sandmann et al., 2011). Thus, AKT2 can modulate the membrane voltage by switching between its modes of an inward or a non-rectifying channel, respectively, and phosphorylation acts as a tool for fine-tuning (Deeken et al., 2000; Michard et al., 2005b). Depending on the cellular context, the phosphorylation status of the AKT2 channels may change, enabling them to drive either inward or outward K^+^ fluxes (Michard et al., 2005a). Of note is that ABA reduces *SKOR* expression in the xylem while increasing that of *AKT2* in the phloem (Pilot et al., 2003). This dual effect reduces K^+^ transport to the shoots and increases delivery of K^+^ to the roots *via* the phloem, thus helping in maintaining a low osmotic potential in water-deprived and salt-stressed roots.

The protein kinase(s) targeting AKT2 remains to be identified. The presence of PKA-like activity in plant cells is poorly documented and awaits confirmation. Held et al., 2011, demonstrated the association of AKT2 with CIPK6 and CBL4 and the effect of this assembly on macroscopic AKT2 currents. However, in contrast to the AKT1-CIPK-CBL complexes, no phosphorylation event could be detected *in vitro*. Instead, it was proposed the Ca^2+^-dependent targeting of AKT2 to the plasma membrane depended solely on the physical interaction of AKT2 with CIPK6/CBL4. The plasma membrane localized receptor-like pseudo-kinase MRH1/MDIS2 also interacts with AKT2 (Sklodowski et al., 2017). MRH1 appears to be essential for AKT2 function since the phenotype of *mrh1-1* and *akt2* knockouts were similar in energy-limiting conditions. However, electrophysiological analyses showed that MRH1 did not affect AKT2 transport. Moreover, the putative kinase domain of MRH1 lacks essential sites that are indispensable for a functional kinase suggesting that MRH1 is a pseudo-kinase and that MRH1 and AKT2 are parts of a bigger protein complex in which MRH1 might help to recruit other unknown partner(s), that might post-translationally regulate AKT2 (Sklodowski et al., 2017).

Because phloem comprises living cells, the K^+^ content in phloem is inherently high (50–150 mM) and the pH is near neutrality (Ahmad and Maathuis, 2014). Consequently, secondary transport in and out the sieve tubes cannot be extensively linked to the H^+^-motive force. K^+^ and photoassimilates are loaded together in source tissues and downloaded in sinks. At source tissues, H^+^-coupled sucrose transporters load the sugar into the phloem. The influx of H^+^ leads to membrane depolarization of the phloem cells, thereby reducing the driving force for further sucrose loading. Depolarization is prevented by the release of K^+^ by AKT2 (Deeken et al., 2002). Thus, K^+^ in the phloem stimulates sugar loading into the phloem sap. Moreover, the post-translational regulation of AKT2 channel activity described above might play a role in the fine-tuning of photoassimilate distribution within the plant by way of controlling the membrane potential through the modulation of K^+^ fluxes into the phloem (Michard et al., 2005a; Gajdanowicz et al., 2011). Accordingly, the expression level of *AKT2* increases in the presence of light and CO_2_ assimilates (Deeken et al., 2000; Lacombe et al., 2000b). In summary, through reversible post-translational modifications, AKT2 taps a “potassium battery” providing additional energy for transmembrane transport processes besides energization by the plasma membrane H^+^-ATPase.

Members of the KT/HAK/KUP family, e.g. AtKUP7 and OsHAK5, have been proposed to facilitate long-distance K^+^ transport from root to shoot, presumably by mediating K^+^ uptake into the xylem parenchyma cells (Yang et al., 2014; Han et al., 2016). This function of KT/HAK/KUP transporters would be relevant under K^+^ deprivation, when apoplastic K^+^ levels could be below the operational range of channels. In rice, OsHAK1 (as well as OsAKT1) seems also to participate in the root-to-shoot transfer of K^+^ and grain yield (Chen et al., 2015); however, it is likely that OsHAK5 dominates K^+^ translocation from roots to shoots at low-K^+^ supply. A role in K^+^ transfer from root to shoot has been also proposed for OsHAK21 under salt stress condition, but not under K^+^ starvation situations (Shen et al., 2015). The *Vitis* VvKUP1, VvKUP2, and the voltage-gated channels VvK1.1 and VvK1.2 have been described to function in K^+^ accumulation during grape berry development (Davies et al., 2006; Cuellar et al., 2010; Cuellar et al., 2013).

As mentioned earlier, HKT channel-like proteins are primarily involved in Na^+^ fluxes both in roots (monocots) and vascular bundles (monocots and dicots) (Hamamoto et al., 2015). However, they often have a significant impact in maintaining high K^+^/Na^+^ ratio in aerial parts during salinity stress and genetic diversity in HKT proteins meditating long-distance transport of Na^+^ and K^+^ have a great impact on the salt tolerance of cereals (Ren et al., 2005; Munns et al., 2012; Zhang et al., 2018). Notwithstanding the above generalization, the class-II HKT transporter of maize ZmHKT2 used K^+^ as the preferred substrate, was mainly expressed in the root stele, and regulated root-to-shoot K^+^ delivery. Domain-swapping between natural variants of ZmHKT2 imparting contrasting salt tolerance indicated that the amino acid variant A130G accounted for differential rates of K^+^ transport to shoots (Cao et al., 2018). On the other hand, mutants in *AtHKT1;1* of *Arabidopsis* and *OsHKT1;5* of rice accumulated significantly less K^+^ in shoots and xylem sap under salinity stress despite the fact that these transporters are Na^+^-selective (Uozumi et al., 2000; Sunarpi et al., 2005; Kobayashi et al., 2017). The uptake of Na^+^ into xylem parenchyma cells by AtHKT1;1 and OsHKT1;5 possibly causes depolarization of the plasma membrane that triggers K^+^ secretion into the xylem vessel *via* outward-rectifying K^+^ efflux channels (Hauser and Horie, 2010). Support for this proposal still requires the analysis of genetic interactions between *hkt1*-like and *skor*-like mutants.

K^+^ is the preferred counter ion for root-to-shoot translocation of NO_3_^−^ in the xylem of crops and *Arabidopsis* (Engels and Marschner, 1993; Zhang et al., 2010; Rodenas et al., 2017). NRT1.5, a member of the Nitrate Transporter 1/Peptide Transporter Family (NPF7.3), is important for the NO_3_^−^-dependent K^+^ translocation in *Arabidopsis* (Lin et al., 2008; Drechsler et al., 2015; Meng et al., 2016). Lack of NRT1.5 resulted in K^+^ deficiency in shoots under low NO_3_^−^ availability, whereas the root elemental composition was unchanged (Lin et al., 2008; Drechsler et al., 2015). Mutant analyses revealed that both NRT1.5 and SKOR contributed additively to K^+^ translocation; SKOR activity was dominant under high NO_3_^−^ and low K^+^ supply, and NRT1.5 was required under low NO_3_^−^ (Drechsler et al., 2015; Li et al., 2017). Accordingly, the *Arabidopsis* mutant *lks2*, unable to grow in low-K^+^, is a loss-of-function mutant in *NRT1.5* (Li et al., 2017). Together, these data indicate that NRT1.5 facilitates K^+^ release out of root parenchyma cells and K^+^ loading into xylem vessels. NRT1.5 is a plasma membrane protein that in *Xenopus* oocytes behaved as a low-affinity, pH-dependent bidirectional nitrate transporter (Lin et al., 2008). Surprisingly, NRT1.5 has also been shown to release K^+^ from *Xenopus* oocytes and yeast in a pH-dependent manner, and has been proposed to function as a K^+^/H^+^ antiporter (Li et al., 2017. If confirmed by additional research, the data of Li et al. (2017) imply that the linkage between NO_3_^−^ and K^+^ transport is more intimate than the mere balancing of charges as previously thought.

## CO-Regulation of K^+^ and Nitrogen Uptake

Plants take up numerous mineral nutrients from the soil; some of them are essential (as K^+^ or NO_3_^−^), while others can be toxic at high concentrations (as Na^+^ or NH_4_^+^). Adaptive responses to varying mineral nutrient conditions in the soil, particularly low-nutrient environments, involve multiple signaling pathways whose integration allows plants to grow and adjust their development to each specific nutritional situation (Kellermeier et al., 2014). Thus, changes in the concentration of one nutrient trigger a signaling cascade that modify not only the amount, localization, and/or activity of this nutrient-specific transporter/channel, but also transporters/channels related with other nutrients. N-K interactions are important for root architecture (Kellermeier et al., 2014). In the previous section, we have discussed the linkage of NO_3_^−^ and K^+^ in long-distance transport. Here, we present the recent knowledge gained about the coordinated regulation of K^+^ and NO_3_^−^ uptake and nutrition.

K^+^ starvation is required for triggering high-affinity HAK5-mediated K^+^ uptake in roots of *Arabidopsis* and tomato. However, limitation of K^+^, N, or P, all induced hyperpolarization of the plasma membrane of root cells and enhanced *HAK5* transcription (Rubio et al., 2014), a response that could be due to maintenance of electrical balance since single N and P starvation, probably resulting in lower NO_3_^−^ and PO_4_^3−^ contents, led to a concomitant reduction of the K^+^ content (Kellermeier et al., 2014). Alternatively, the transport of a nutrient could become inhibited if another nutrient is limiting growth (Nieves-Cordones et al., 2019). In line with this, NO_3_^−^, PO_4_^3−^, and SO_4_^2−^ deficiencies reduced root K^+^ uptake (Rodenas et al., 2017). Furthermore, comparison of the transcriptional responses to single or multiple nutrient deprivations showed that N starvation had a dominant effect over P and K starvation. In other words, the transcriptional landscape of combined K^+^ and N limitation was mainly driven by the N-starvation response.

For most plants, nitrate (NO_3_^−^) and ammonium (NH_4_^+^) are the two major nitrogen sources (Crawford, 1995; Gazzarrini et al., 1999). In general, in aerobic soil conditions, nitrate is the primary nitrogen source, while under anoxic conditions ammonium is (Ho and Tsay, 2010). To be assimilated, NO_3_^−^ has to be taken up from the soil and converted into ammonium by nitrate and nitrite reductases, and then incorporated into amino acids *via* the glutamine-synthetase and glutamate-synthase (GS-GOGAT) pathway. Therefore, ammonium is the preferred nitrogen source in plants, but ammonium uptake *via* the roots is tightly controlled because elevated ammonium concentrations in the cytosol are toxic (Straub et al., 2017). The mechanisms underlying ammonium toxicity are not fully understood, but acidification of the external environment, disruption of the acid/base balance, and the energy lost exporting excess ammonium may be key factors. NO_3_^−^ and NH_4_^+^ uptake systems involve different families of proteins and a complex regulation not fully understood yet. Here, we will focus on *Arabidopsis* dual-affinity NO_3_^−^ transporter AtNRT1.1 (Nitrate Transporter 1, also called AtCHL1 and AtNPF6.3) and high-affinity NH_4_^+^ transporters AtAMT1, because they share part of their post-translational regulation with AtAKT1 and AtHAK5.

**Figure 4:**
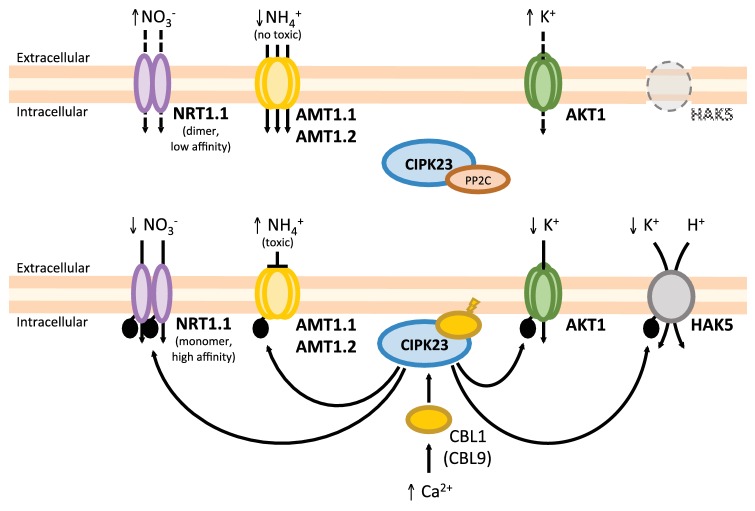
Concerted regulation of nitrate, ammonium, and potassium transport. *Top*, under nutrient sufficiency, the nitrate transporter NRT1.1 operates in the low-affinity mode, AMTs transport any NH_4_^+^ present in the soil solution as the preferred N source, and AKT1 ensures K^+^ influx down its electrochemical gradient. *Bottom*, upon nutrient scarcity, the N-myristoylated and Ca^2+^-bound CBL1/9 recruit to the plasma membrane and activate the protein kinase CIPK23 that in turn phosphorylates and activates the high-affinity mode of transport of NRT1.1 and HAK5, and stimulates K^+^ influx through AKT1. CIPK23 is also able to inhibit AMTs when the NH_4_^+^ is at toxic levels.

The CIPK23/CBL1,9 protein kinase complex is key factor in the coordination of plant nutrition, regulating iron, NO_3_^−^, and K^+^ uptakes (Ho et al., 2009; Ragel et al., 2015; Straub et al., 2017; Dubeaux et al., 2018). The transport and regulatory protein AtNRT1.1 is involved in both high-affinity and low-affinity nitrate uptake. Unphosphorylated AtNRT1.1 is a low-affinity nitrate transporter working as a dimer, and its phosphorylation by CIPK23/CBL1,9 leads to dimer dissociation. Phosphorylated AtNRT1.1 monomers show a higher nitrate affinity than the dimers (Ho et al., 2009; Sun et al., 2014). On the other hand, AtAMT1 ammonium transporters work as trimers and the phosphorylation by CIPK23/CBL1 (and not CBL9) of a single monomer exhibits an allosteric effect, leading to the cooperative closure of all three pores in the trimer (Straub et al., 2017). Together, these data indicate that CIPK23 and CBL1 are major regulators of NO_3_^−^, K^+^, and NH_4_^+^ homeostasis in *Arabidopsis*. As Figure 4 shows, under K^+^- and NO_3_^−^-sufficient and NH_4_^+^-moderate (non-toxic) conditions, CIPK23 is in the cytoplasm and inactive because of the interaction with protein phosphatases of the PP2C family (Lee et al., 2007; Cherel et al., 2014). In this situation, AtNRT1.1 will work as dimeric low-affinity NO_3_^−^ transporter and AtAMT1 trimers will be active, unphosphorylated AtAKT1 will be less active and *AtHAK5* will be transcriptionally repressed. Thus, N demand is covered by NO_3_^−^ and NH_4_^+^ uptake, and K^+^ is supplied by AKT1. Under K^+^ and/or NO_3_^−^ low concentrations or toxic NH_4_^+^ conditions, AtCIPK23 is recruited by AtCBL1 or AtCBL9 to the plasma membrane, which allows the phosphorylation of CIPK23 target transporters. Phosphorylated AtAMT1 trimers are inactive and N demand is covered by high-affinity NO_3_^−^ transport through phosphorylated AtNRT1.1 monomers. Phosphorylated AtAKT1 will enhance K^+^ influx, serving as a balancing counter ion for NO_3_^−^. If the K^+^ concentration is low enough, *AtHAK5* will be transcribed and the AtHAK5 protein activated by CIPK23/CBL1,9 complex.

Formation of second-order lateral roots in *Arabidopsis* was significantly stimulated by K^+^ starvation, but low-N inhibited this effect of low-K^+^. Mutation of *AKT1* or *CIPK23* also cancelled the formation of lateral roots under low-K^+^ (Kellermeier et al., 2014). These nitrate-specific effects occurred over a concentration range that triggers phosphorylation of NRT1.1 by CIPK23. These results suggest that N and K^+^ availability determines root architecture and that CIPK23 serves as the regulatory node acting through both AKT1 and NRT1.1.

It is noteworthy that AtNRT1.1 has been described as a transceptor (i.e., a transporter that is also serving as sensor/receptor for its substrate) (Ho et al., 2009; Wang et al., 2009), but its uptake activity is not required for the sensing function. As a NO_3_^−^ sensor, when plants are exposed to high concentrations of NO_3_^−^, dephosphorylated AtNRT1.1 leads to the primary NO_3_^−^-response, consisting in the rapid expression of nitrate assimilatory enzymes and nitrate transporters to prepare the plant to assimilate NO_3_^−^. In response to low concentrations of NO_3_^−^, CIPK23 phosphorylates AtNRT1.1 that in turn will down-regulate the primary response. Therefore, the phosphorylation status of AtNRT1.1 not only switches the transport modes, but also induces different levels of primary nitrate response, and CIPK23 works as a negative regulator of the primary nitrate response. How the phosphorylation status of AtNRT1.1 modulates the transcriptional levels remains unknown (Ho and Tsay, 2010). Transcriptional responses to low-N showed a strong additive effect by low-K^+^ (Kellermeier et al., 2014). Moreover, NO_3_^−^ deprivation increased *AtHAK5* and *LeHAK5* expression to a similar level than K^+^ deprivation (Rubio et al., 2014). The transcription factors and signaling pathways involved in this coordinated response to nutrient deprivation have not been characterized.

Finally, as mentioned before, the transporters of the NPF group AtNRT1.5 and AtNRT1.8 may transport K^+^ besides or instead NO_3_^−^ (Li et al., 2017). AtNRT1.5 plays a crucial role in K^+^ translocation from root to shoot and is involved in the coordination of K^+^/NO_3_^−^ distribution in plants (Drechsler et al., 2015). Furthermore, the putative orthologs of AtNRT1.5 from rice (OsNPF7.9) and maize (ZmNPF7.10) can also function as K^+^ efflux transporters (Li et al., 2017), suggesting that the K^+^ transport function of NRT1.5-like proteins is conserved in vascular plants. These results lay an additional layer to the coordinated transport of N and K^+^ besides the co-regulation by CIPK23 of NO_3_^−^- and K^+^-specific transporters.

## Conclusions and Perspectives

Although plant responses to K^+^ starvation are well documented at the physiological and transcriptional levels, the sensing and regulatory mechanisms underlying these changes need to be clarified. Despite all the progress made regarding the regulation of individual K^+^ transporters and the signaling pathways involved, how depletion of cellular K^+^ is sensed remains poorly understood. Hyperpolarization of the plasma membrane under limiting K^+^ seems to be a key factor in the transcriptional activation of genes encoding high-affinity K^+^ uptake, but other early signaling events activating the known cascades of phytohormones, ROS production, and post-translational modifications of K^+^ transport proteins remain unclear. It has been proposed that AKT1 may act as a K^+^ sensor in the root architecture response to nutrient supply, possibly by linking auxin transport to plasma membrane potential (Kellermeier et al., 2014). Moreover, it is also worth noting that transient and localized cytosolic K^+^ spikes have been proposed to work, together with Ca^2+^ and ROS waves, as messengers that signal and shape plant adaptive responses to stress (Shabala, 2017). Nonetheless, fundamental questions regarding the sensing of and responses to K^+^ deprivation are of central importance for plant nutrition and deserve additional research. The availability of novel genetically encoded K^+^ sensors (Bischof et al., 2017) that could be targeted to various cellular compartments of predefined cells and tissues will be powerful tools to monitor the dynamics of cellular K^+^ with unprecedented spatiotemporal resolution.

## Author Contributions

All authors have contributed to literature search, discussion, and writing of the manuscript. PR and JP assembled all sections. PR and FQ prepared the Figures. All authors checked and approved the manuscript.

### Conflict of Interest Statement

The authors declare that the research was conducted in the absence of any commercial or financial relationships that could be construed as a potential conflict of interest.
